# Supplementary material for: Sox17 drives functional engraftment of endothelium converted from non-vascular cells
Source: Nat Commun. 2017 Jan 16;8:13963. doi: 10.1038/ncomms13963 (PMC5260855; doi:10.1038/ncomms13963)
Supplement: Supplementary Information — Supplementary Figures 1–5, Supplementary Table 1–7, Supplementary Methods. [file ncomms13963-s1.pdf]

SUPPLEMENTARY INFORMATION

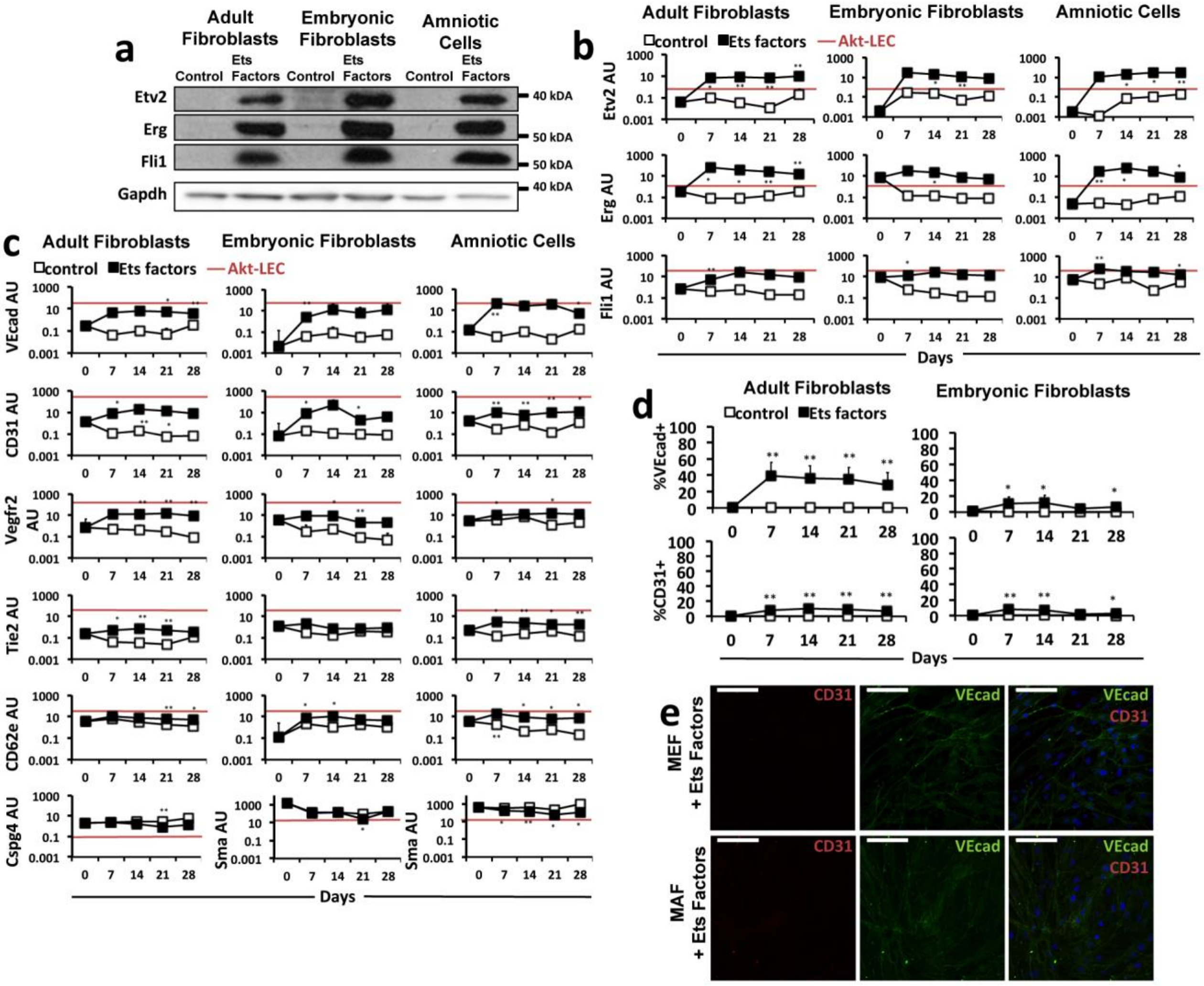

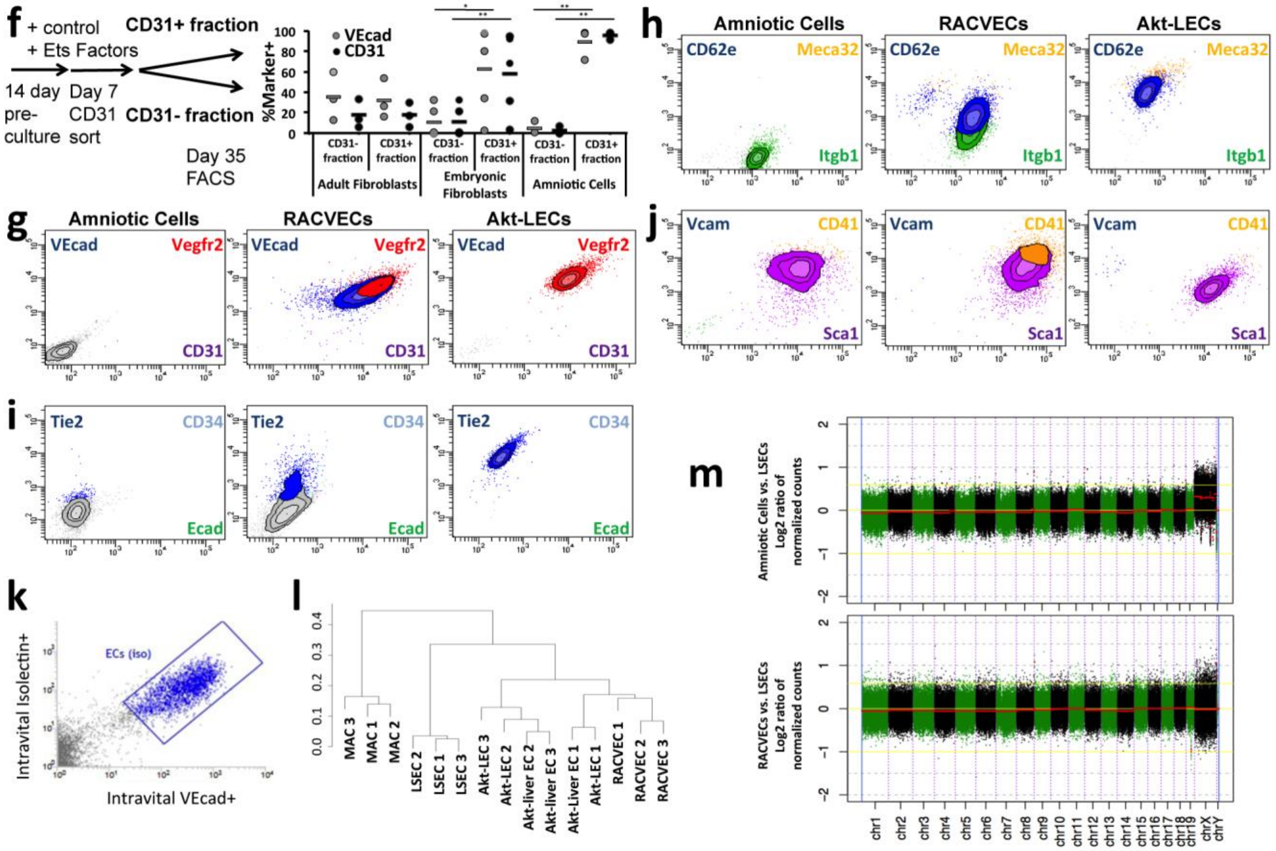

Supplementary Figure 1. Adult and embryonic fibroblasts do not efficiently convert to EC-like cells. a, Western blotting showing expression of lentivirally-introduced Ets transcription factors Etv2, Erg, and Fli1 at day 7. **b**, QPCR showing expression of Etv2, Erg, and Fli1 transcript levels. **c**, QPCR showing expression of transcript levels (n=6). The red line indicates the average level observed in Akt-LECs (n=7). **d**, Surface expression of VEcad and CD31 on control and Ets transcription factor–infected adult fibroblasts and embryonic fibroblasts analyzed by flow cytometry (n=6 for adult fibroblasts, n=5 for embryonic fibroblasts). **e**, Surface expression of VEcad and CD31 on MAFs and MEFs after day 28 as detected by fluorescent microscopy. Scale bars = 100 μM **f**, Cells were grown in conversion conditions for 7 days and then the CD31-positive and negative fractions were isolated by FACS. Surface expression of VEcad and CD31 were subsequently analyzed by flow cytometry at day 35 (n=3). **g-j**, Representative plots of EC protein expression for MACs, RACVECs, and Akt-LECs. For each plot, the x- and y-axes are labeled and

cells positive for a third maker, indicated in the upper right corner, are colored in red (**g**), orange (**h**), light blue (**i**), and orange (**j**). AU = arbitrary units, bar heights indicate means, error bars indicate standard deviations among biological replicates, asterisks indicate $p < 0.05$ and double asterisks indicate $p < 0.01$ two-sided t-tests assuming normal distribution. **k,** Sample FACS plot of LSEC purification by intravital anti-VEcad and isolectin staining. **l**, Hierarchical clustering based on FPKMs of genes within the “angiogenesis” GO category for MACs, RACVECs, directly isolated LSECs, Akt-liver ECs, and Akt-LECs. **m** Plots reads counts are shown for the indicated cell types over directly purified LSECs. The yellow line at Y=0 represents equal amounts of read counts, the slashed yellow line at Y=0.58 represents one duplication, and the dotted yellow line at Y= - 1 indicates a loss of one copy. The segmentation line is indicated in red. The chromosomes are shown in alternating colors and labeled by number.

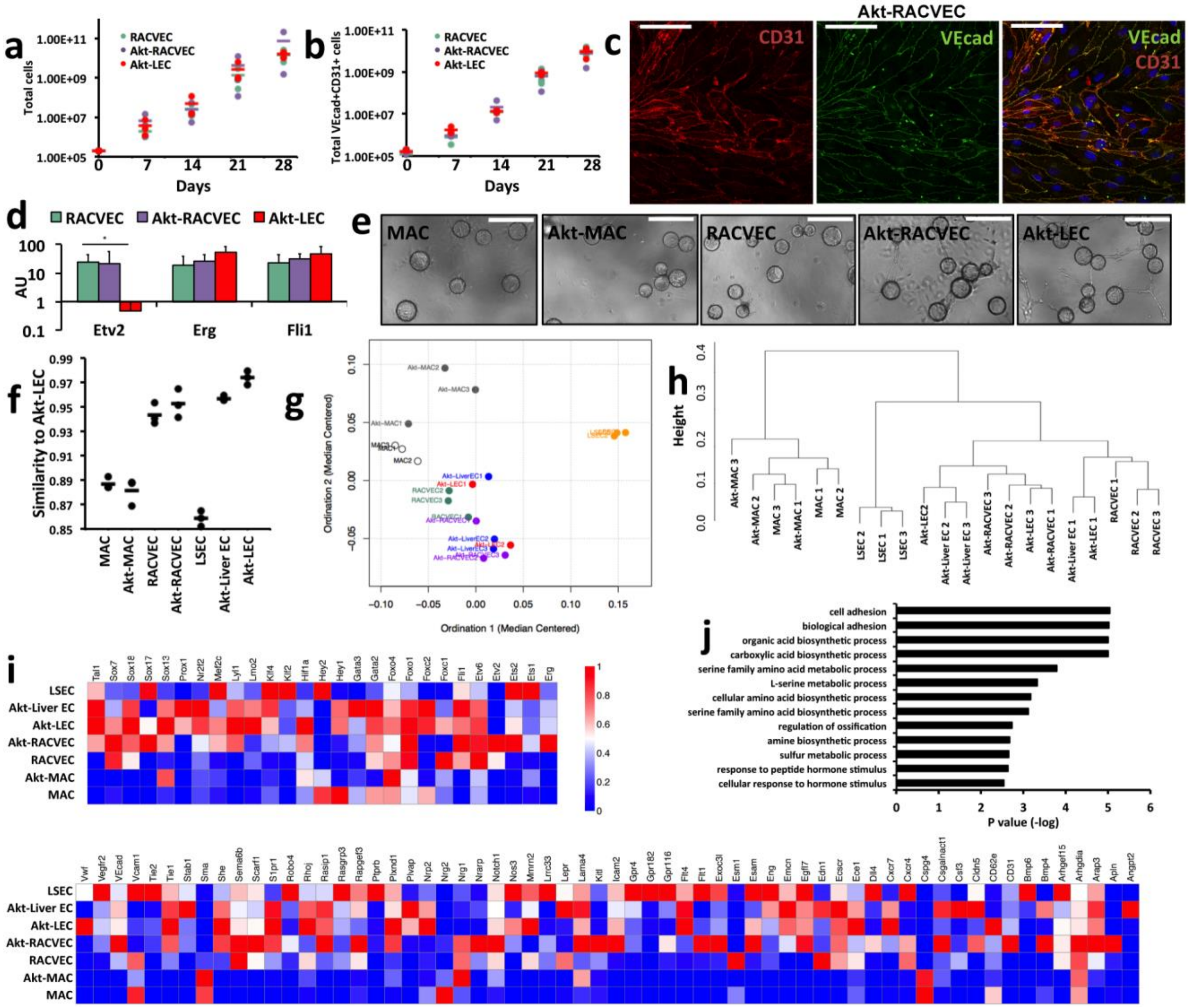

Supplementary Figure 2. Constitutive Akt-signaling endows RACVECs with EC functions. **a**, RACVECs, Akt-RACVECs and Akt-LECs were seeded at equal numbers and grown in EC growth conditions. At the indicated time points, cells were counted and **b**, the percentages of cells that were VEcad^{+}CD31^{+} were determined by flow cytometry (n=3). **c**, Surface expression of VEcad and CD31 on Akt-RACVECs as detected by fluorescent microscopy. Scale bars = 100 μM **d**. QPCR detecting transcripts of Etv2, Erg and Fli1 RACVEC, Akt-RACVEC, and Akt-LEC (n=6). **e**, Representative images used to calculate percentages of connected beads for MAC, Akt MAC, RACVEC, Akt-RACVEC, Akt-LEC. Scale bars = 500μM. **f**, An average Akt-LEC sample was calculated and 1-Pearson correlations between expression profiles were calculated to represent

the proximity of each sample to the average Akt-LEC sample. **g**, Principal component analysis based on FPKMs of indicated samples. Dots represent individual isolates and are colored by sample type. **h**, Hierarchical clustering based on FPKMs of genes within the “angiogenesis” GO category for MACs, Akt-MACs, RACVECs, Akt-RACVECs, cultured Akt-liver ECs and Akt-LECs. **i**, Heatmaps of representative LSECs, Akt-liver ECs, Akt-LECs, Akt-RACVECs, RACVECs, Akt-MACs, and MACs with selected EC genes. Colors reflect Z-scores of individual isolates with blue representing 0 and red representing 1, the maximum FPKM value for a given transcript. **j**, GO term analysis using the set of genes in which FPKM values were decreased by more than log2, $P < 0.05$, for Akt-RACVECs versus RACVECs. For all panels, bar heights indicate means, error bars indicate standard deviations among biological replicates performed at different times, asterisks indicate $p < 0.05$ and double asterisks indicate $p < 0.01$ two-sided t-test assuming normal distribution.

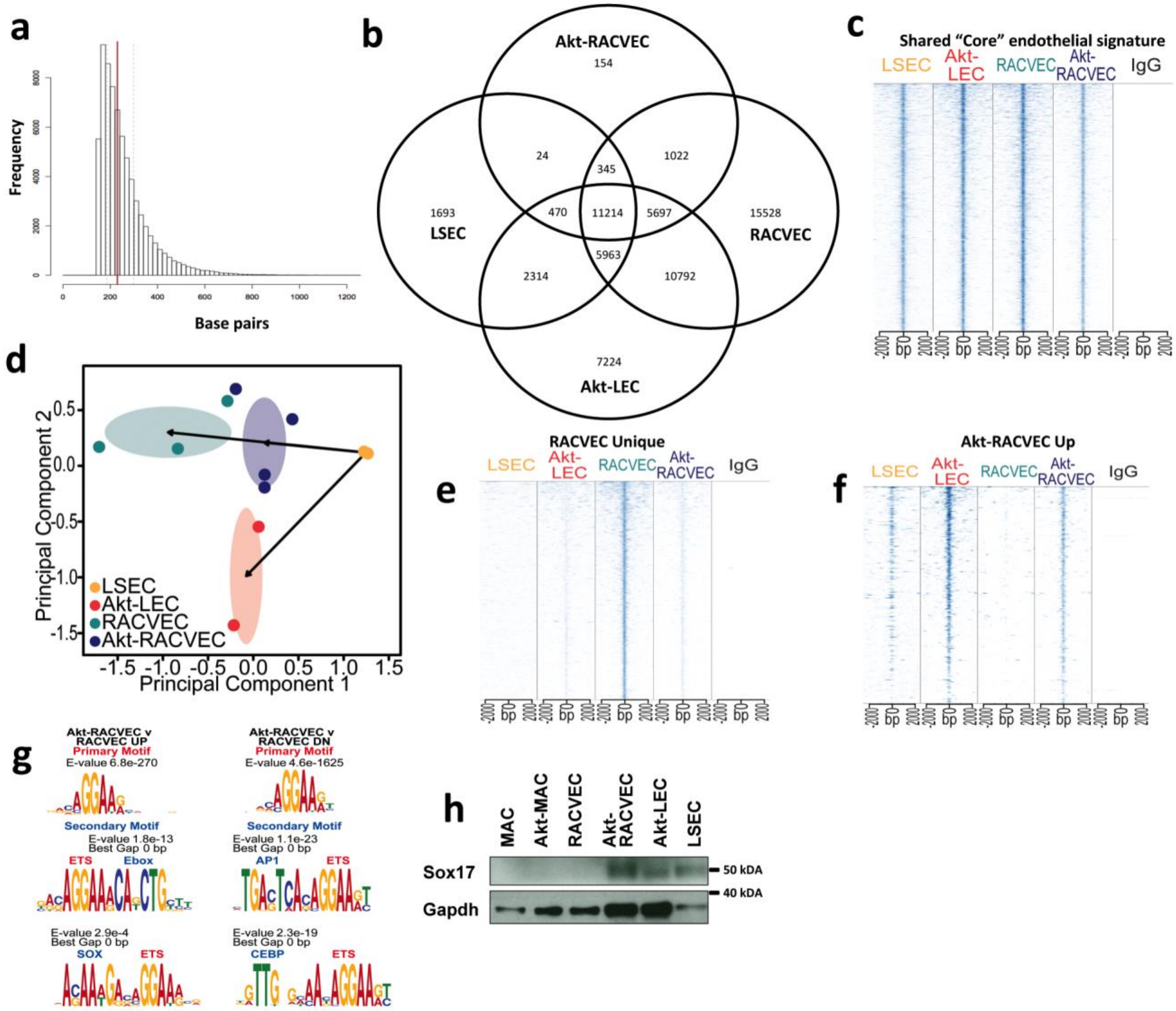

Supplementary Figure 3. The genomic targets of Fli1 are altered in the presence of constitutive Akt-signaling. a, Histogram depicting the width of all Fli1-bound regions. The red line indicates the median width and the dotted grey lines are the 25th and 75 percentiles. **b,** Simplified Venn diagram with numbers of Fli1-bound regions, compared to IgG, contained in each group. This figure is not to scale. **c**, Heatmap of Fli1-binding signals at each site belonging to the set of "Core" DBRs (-2500 to + 2500 bp) shown in rows. **d,** Principal component analysis based on normalized Fli1-binding logFC. Dots represent individual isolates and are colored by sample type. The colored ovals represent the means of each cell type and the size is determined by the standard deviation along the two principle components. The arrows indicate the dissimilarities

between groups. **e,** Heatmap of Fli1-binding signals at each site in the RACVEC unique set. **f,** Heatmap of Fli1-binding signals at each site in the set composed of regions more strongly bound in Akt-RACVECs compared to RACVECs, known as Akt-RACVEC Up. **g,** Enrichment of secondary motifs representative of TF families is shown for the indicated datasets. **h**, Western blotting showing Sox17 protein in MAC, Akt-MAC, RACVEC, Akt-RACVEC, Akt-LEC, and LSEC samples.

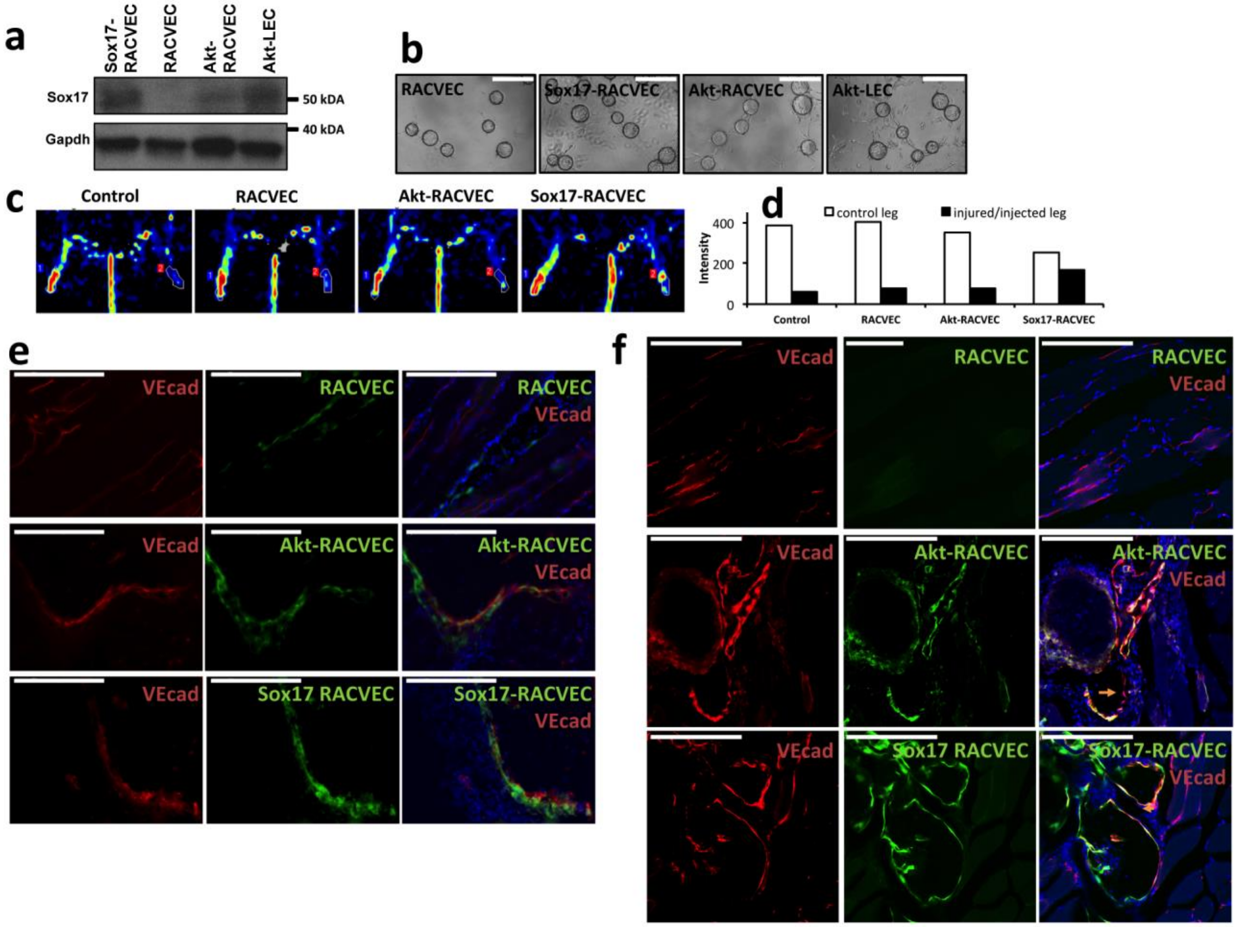

Supplementary Figure 4. Sox17 enhances conversion and endows RACVECs with EC functions. a, Western blotting showing Sox17 protein in Sox17-RACVECs, RACVECs, Akt-RACVECs, and Akt-LECs. **b**, Representative image used to calculate percentages of connected beads for Sox17-RACVEC. Scale bars = 500μM. **c**, Representative Doppler images from day 14 of mice that underwent unilateral femoral artery excision and were injected with PBS, RACVEC, Akt-RACVEC, and Sox17-RACVEC. **d**, Samples intensity values derived from perfusion images shown in Supplementary Figure 4c. **e**, Mice that underwent unilateral artery excision and RACVECs, Sox17-RACVECs, or Akt-RACVEC injection were injected with fluorescently labeled anti-VEcad antibody and sacrificed. Images of sections of mouse limbs 1 day after surgery and injection. Scale bars = 200μM. **f**, Images of sections of mouse limbs 2 months after surgery and injection. Scale bars = 200μM.

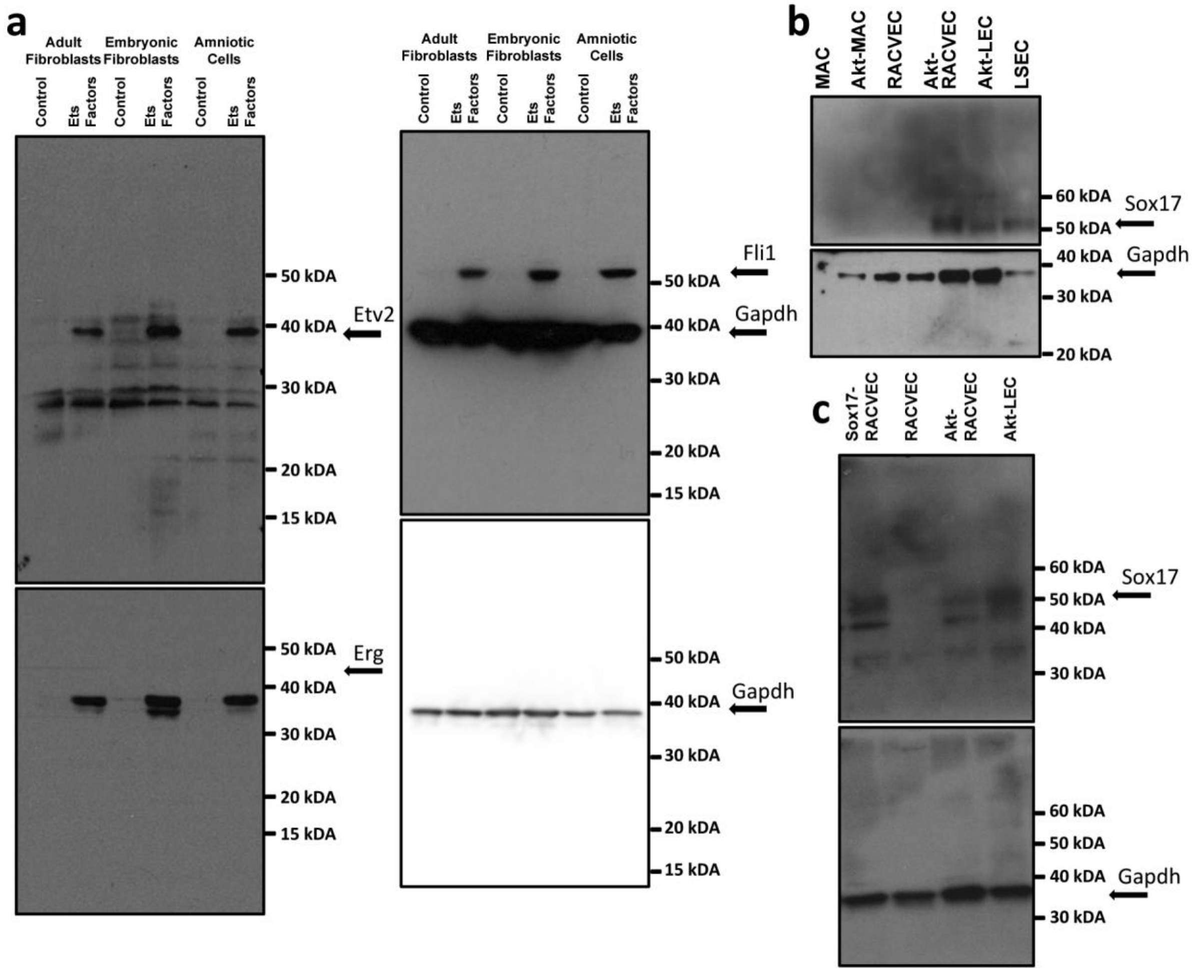

Supplementary Figure 5. Uncropped Western blots. a. Uncropped versions of Western blots shown in Supplementary Figure 1a. Note that Fli1 and Gapdh protein were detected on the same membrane. The highly exposed image was used to determine Fli1 protein levels while a briefly exposed image, shown below, was used to visualize Gapdh levels. b. Uncropped versions of Western blots shown in Supplementary Figure 3h. c. Uncropped versions of Western blots shown in Supplementary Figure 4a.

Supplementary Table 1.

	Akt-Liver EC 1	Akt-Liver EC2	Akt-Liver EC3	Akt-LEC 1	Akt-LEC 2	Akt-LEC 3	MAC 1	MAC 2	MAC 3	Akt-MAC 1	Akt-MAC 2	Akt-MAC 3
Sbsn	18	4	3	17	2	1	7	23	48	154	13	73
Pecam1	635	926	576	373	778	925	0	0	4	2	13	11
Kdr	427	1298	1171	303	1430	441	2	3	12	14	9	3
Flt4	406	2425	1579	525	1705	75	11	0	7	7	9	81
Ube2c	318	76	275	457	436	503	21	97	53	85	39	54
Top2a	215	56	178	415	200	363	24	101	43	69	34	27
Ckap2	132	40	83	169	117	157	43	72	55	57	31	26
Inhbb	159	179	166	134	19	352	5	33	53	298	11	29
Cks2	91	23	73	100	74	55	16	23	22	33	12	17
Tk1	91	23	90	136	81	159	20	51	13	29	14	17
Tnc	1695	3417	2028	3302	40	856	624	270	232	523	412	145
Nts	110	92	1124	110	4960	16	0	0	0	8	0	0
Prc	156	31	111	243	190	201	22	45	38	69	29	24
Mk176	91	30	57	175	102	115	7	39	15	26	12	10
Itgb3	413	535	208	201	705	290	8	4	122	40	11	23
CD276	55	189	195	54	11	72	144	114	310	87	79	30
Etv4	70	137	129	104	31	28	71	90	83	32	28	51
Tnfrsf9	0	0	8	0	1	0	0	0	0	0	0	0
Kcne3	35	97	41	10	0	0	0	0	0	0	0	0
Ubd	0	2	0	0	17	13	0	39	0	0	0	0
Prnd	30	66	24	19	10	110	3	0	0	5	5	2
Apln	3167	465	46	1803	45	4800	225	85	44	17	38	2
Pgf	625	348	158	181	69	539	15	0	37	4	10	4
Ptprn	208	800	148	311	14	60	158	20	440	201	106	163
CD109	66	93	24	58	36	155	175	95	334	241	57	10
Ank	161	79	147	100	61	9	277	627	316	178	226	124
Col8a1	97	4	1	132	49	629	44	541	60	130	281	134
Antxr1	58	69	70	233	34	136	110	169	196	166	74	66
CD248	67	2	1	160	2	13	373	816	202	156	39	38
Rasd	0	4	0	0	41	0	1	0	5	2	10	1
Plxndc1	1	0	1	1	0	0	3	1	3	3	0	0
Arhgef17	43	22	19	105	5	7	159	134	156	98	85	114
Gpr124	160	281	155	343	207	299	249	464	344	258	72	24
Tns3	222	108	71	264	10	81	120	109	148	216	340	317
Plxndc2	21	1	1	161	0	0	454	126	154	102	189	115

	RAC VEC 1	RACVEC 2	RACVEC 3	Akt-RACVE 1	Akt-RACVEC 2	Akt-RACVEC 3	LEC 1	LEC 2	LEC 3
Sbsn	4	30	134	9	0	3	5	3	21
Pecam1	82	27	6	1127	2353	1562	1690	2391	1287
Kdr	206	97	179	1034	1016	1198	121	692	394
Flt4	165	80	113	193	363	111	139	447	266
Ube2c	368	106	270	356	372	127	149	77	122
Top2a	201	74	162	341	298	178	109	64	93
Ckap2	128	64	115	85	103	46	89	46	48
Inhbb	185	66	663	617	977	311	127	33	48
Cks2	74	27	37	74	83	28	28	34	36
Tk1	96	31	74	171	119	75	29	18	29
Tnc	481	490	382	277	156	6	804	1633	1540
Nts	26	13	4	9	142	2	19	75	67
Prc	162	45	109	109	105	72	85	41	81
Mk176	83	24	44	79	73	42	55	33	42
Itgb3	326	96	118	331	564	562	277	1207	675
CD276	87	120	204	146	206	50	66	119	111
Etv4	31	34	33	33	77	0	16	48	43
Tnfrsf9	0	0	0	0	0	0	0	0	0
Kcne3	2	5	1	0	76	0	6	38	31
Ubd	17	135	3	0	0	29	115	49	29
Prnd	9	4	1	17	133	36	780	1207	640
Apln	503	426	883	1465	4165	1725	1513	1909	662
Pgf	49	66	222	101	907	15	747	131	158
Ptprn	138	129	101	175	146	4	196	298	454
CD109	142	69	109	121	176	114	115	81	390
Ank	114	158	70	40	69	1	55	189	209
Col8a1	1	457	86	71	13	132	428	22	366
Antxr1	56	84	58	60	33	11	167	93	204
CD248	19	478	240	20	1	3	16	23	1175

Rasd	0	0	0	1	1	62	10	0	1
Plxndc1	0	0	0	0	0	0	0	2	0
Arhgef17	89	82	80	27	42	1	50	42	121
Gpr124	201	358	428	205	287	164	219	158	281
Tns3	49	41	71	46	56	21	123	133	227
Plxndc2	0	92	13	50	0	0	51	10	74

Supplementary Table 2.

Table 2 Core vs IgG

Comparison	Motif	E-value	Similar Motifs	Family
FLI1 (vs IgG)	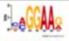	1.2e-1506	ELF5, ELK4, ETS1, FLI1, ERG	ETS
FLI1 (vs IgG)	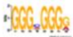	1.3e-112	SP1, EGR1, KLF5	SP1, Kruppel-like, GC-box
FLI1 (vs IgG)	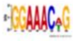	2.6e-72	Foxo1	Fox, ETS
FLI1 (vs IgG)	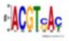	2.2e-44	Arntl, BHLHE41, TFE3	bZIP, bHLH/ Ebox
FLI1 (vs IgG)	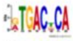	1.3e-38	FOSL2, JUNB, JUND	AP1
FLI1 (vs IgG)	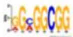	8.0e-32	SP1, SP2, EGR1	SP1, Kruppel-like, GC-box
FLI1 (vs IgG)	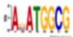	3.4e-21	YY1, E2F2, E2F3	YY1, E2F
FLI1 (vs IgG)	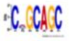	3.8e-20	Myog, Tcf12	bHLH, Ebox
FLI1 (vs IgG)	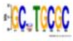	1.1e-16	NRF1	NRF
FLI1 (vs IgG)	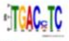	1.3e-13	RARA, NR2F	RAR
FLI1 (vs IgG)	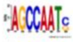	4.5e-10	NFYB	CAAT-box
FLI1 (vs IgG)	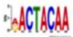	7.1e-10		
FLI1 (vs IgG)	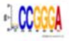	8.4e-10	E2F4	E2F
FLI1 (vs IgG)	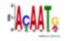	6.2e-8	SOX8, SOX9, Sox17	Sox
FLI1 (vs IgG)	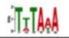	6.2e-8		?Hox

Supplementary Table 3.

Table 3 LSEC vs IgG

Comparison	Motif	E-value	Similar Motifs	Family
Fli1 vs IgG	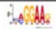	9.4e-1584	ELF5, ETS1, ERG	ETS
Fli1 vs IgG	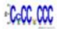	1.3e-133	SP1, EGR1, KLF5	SP1, Kruppel-like, GC-box
Fli1 vs IgG	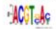	1.7e-46	Arntl, BHLHE41, TFE3	bZIP, bHLH/ Ebox
Fli1 vs IgG	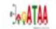	5.9e-43	GATA5, GATA1, Gata4	GATA
Fli1 vs IgG	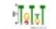	8.1e-29	FOXD2, FOXO4, FOXL1	Fox
Fli1 vs IgG	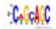	1.5e-22	Tcf12, Myog	bHLH, Ebox
Fli1 vs IgG	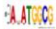	7.7e-19	YY1, E2F2, E2F3	YY1, E2F
Fli1 vs IgG	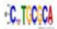	3.7e-18	NRF1	NRF
Fli1 vs IgG	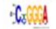	6.8e-18	STAT3, STAT1	STAT
Fli1 vs IgG	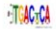	6.8e-17	FOS::JUN, Bach1::Mafk, JDP2	AP1
Fli1 vs IgG	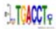	3.9e-14	NR4A2, Nr5a2, NR2C2	Nuclear Receptor
Fli1 vs IgG	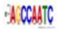	6.2e-11	NFYB	CCAAT-box
Fli1 vs IgG	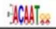	3.4e-7	SOX9, Sox17, Sox6	Sox

Supplementary Table 4.

Table 4 Akt-LEC vs IgG

Comparison	Motif	E-value	Similar Motifs	Family
Fli1 vs IgG		1.4e-4983	EWSR1-FLI1, SPIC	ETS
Fli1 vs IgG		1.1e-173	KLF5, SP1, EGR1	SP1, Kruppel-like, GC-box
Fli1 vs IgG		1.3e-166	FOS, JUND, FOSL1	AP1
Fli1 vs IgG		1.1e-104		?Fox
Fli1 vs IgG		1.2e-96	Foxq1, HOXA13, HOXB13	Fox, Hox
Fli1 vs IgG		1.1e-65	Arntl, SREBF2, BHLHE41	bHLH, Ebox
Fli1 vs IgG		1.8e-37	Nr5a2, ESR2, NR4A2	Nuclear Receptor
Fli1 vs IgG		1.6e-35	SOX8, SOX9, Sox17	Sox
Fli1 vs IgG		1.0e-30	NHLH1, Ascl2, Tcf12	
Fli1 vs IgG		5.3e-25	GATA1::TAL1, GATA5, GATA2	GATA
Fli1 vs IgG		1.4e-17	MEF2A, MEF2C, MEF2B	
Fli1 vs IgG		1.1e-16	NRF1, Hes1	

Supplementary Table 5.

Table 5 RACVEC vs IgG

Comparison	Motif	E-value	Similar Motifs	Family
Fli1 vs IgG		1.7e-5654	EWSR1-FLI1, SPIC	ETS
Fli1 vs IgG		1.5e-610	FOS, FOSL2, JUNB	AP1
Fli1 vs IgG		6.9e-209	SP1, KLF5, EGR1	SP1, Kruppel-like, GC-box
Fli1 vs IgG		1.7e-120		
Fli1 vs IgG		1.2e-105	HOXB13, HOXA13, HOXD13	Hox, ?Fox
Fli1 vs IgG		1.9e-66	EGR1, SP1, SP2	SP1, GC-box
Fli1 vs IgG		3.1e-62	Arntl, SREBF2, TFE3	bHLH, Ebox
Fli1 vs IgG		1.6e-49		

Supplementary Table 6.

Table 6 Akt-RACVEC vs IgG

Comparison	Motif	E-value	Similar Motifs	Family
Fli1 vs IgG		3.8e-2758	EWSR1-FLI1, SPIC, ELF5	ETS
Fli1 vs IgG		4.3e-194	FOS::JUN, MAF::NFE2, Bach1::Mafk	AP1
Fli1 vs IgG		2.1e-142	SP1, KLF5, EGR1	SP1, Kruppel-like, GC-box
Fli1 vs IgG		7.8e-54	Pax2, ESR1, JDP2	Paired-box, Nuclear Receptor
Fli1 vs IgG		2.3e-40	CDX1, HOXB13, HOXD13	Hox
Fli1 vs IgG		1.1e-38	NHLH1, Ascl2, Tcf12	bHLH/E-box, bZIP
Fli1 vs IgG		1.7e-26	Klf1	Kruppel-like
Fli1 vs IgG		1.3e-22	SOX9, Sox6, Sox2	Sox
Fli1 vs IgG		1.6e-20	Creb5, Crem, CREB1	
Fli1 vs IgG		1.5e-18	NRF1	
Fli1 vs IgG		1.8e-13		
Fli1 vs IgG		7.7e-13	YY1, E2F2, E2F3	YY1, E2F
Fli1 vs IgG		7.6e-9	CREB3, Creb3l2, MAX	
Fli1 vs IgG		4.4e-7	NRF1	
Fli1 vs IgG		1.0e-6	TGIF1, MSC, TGIF2	TALE-like

Supplementary Table 7.

Table 7 RACVEC vs Akt-RACVEC

Comparison	Motif	E-value	Similar Motifs	Family
AKT-Up	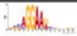	4.6e-272	Gabpa, ELK4, ETV4	ETS
AKT-Up	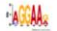	1.7e-49	Fli1, SPIC	ETS
AKT-Up	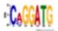	1.3e-05	SPDEF	ETS
AKT-Up	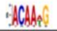	3.3e-06	SOX10, Sox2, Sox6	SOX
AKT-Dn	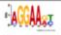	3.1e-1614	Stat4, SPIC, ELF5	ETS
AKT-Dn	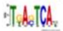	2.0e-207	FOS, JUND, FOSL2	AP1
AKT-Dn	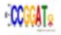	4.6e-38	SPDEF, ETV5, ETV4	ETS
AKT-Dn	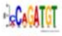	1.4e-13	ZBTB18, TAL1, Bhlha15	E-box (ZnF, bHLH)
AKT-Dn	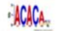	1.5e37		
AKT-Dn	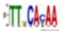	4.4e-34	CEBPA, CEBPB, CEBPE	CCAAT-box
AKT-Dn	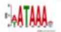	2.7e-28	HOXB13, HOXA13, HOXD13	HOX
AKT-Dn	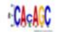	7.3e-10	ZIC1, Gfi1b, ZIC4	C2H2-ZnF
AKT-Dn	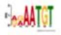	7.5e-9	TEAD1, TEAD4, TEAD3	TEAD (?ETS)
AKT-Dn	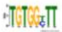	1.3e-6	RUNX1, RUNX3, RUNX2	RUNX
AKT-Dn	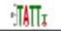	1.6e-6	MEF2A, MEF2B, MEF2D	MADS-box

Supplementary Methods

Primer sequences for QPCR reactions:

Mouse Etv2 F 5’-AACAAGCATCCATGGACCT-3’

Mouse Etv2 F 5’-CTCTGGGAACCCTTTCCAG-3’

Mouse Erg F 5’-ACCTCACCCCTCAGTCCAAA-3’

Mouse Erg R 5’-TGGTCGGTCCCAGGATCTG-3’

Mouse Fli1 F 5’-ATGGACGGGACTATTAAGGAGG-3’

Mouse Fli1 R 5’-GAAGCAGTCATATCTGCCTTGG-3’

Mouse VEcad F 5’-CACTGCTTTGGGAGCCTTC-3’

Mouse VEcad R 5’-GGGCAGCGATTCATTTTTCT-3’

Mouse CD31 F 5’-CTGCCAGTCCGAAAATGGAAC-3’

Mouse CD31 R 5’-CTTCATCCACCGGGGCTATC-3’

Mouse Vegfr2 F 5’-TTTGGCAAATACAACCCTTCAGA-3’

Mouse Vegfr2 R 5’-GCAGAAGATACTGTCACCACC-3’

Mouse CD62e F 5’-ATGCCTCGCGCTTTCTCTC-3’

Mouse CD62e R 5’GTAGTCCCGCTGACAGTA-3’

Mouse Tie2 F 5’-ATGTGGAAGTCGAGAGGCGAT-3’

Mouse Tie2 R 5’-CGAATAGCCATCCACTATTGTCC-3’

Mouse Sma F 5’-GTCCCAGACATCAGGGAGTAA-3’

Mouse Sma R 5’-TCGGATACTTCAGCGTCAGGA-3’

Mouse Gapdh F 5’-AAATGGTGAAGGTCGGTGTGAACG-3’

Mouse Gapdh R 5’-GGTCAATGAAGGGGTCGTTGATGG-3’

Mouse Sox4 F 5’-GACAGCGACAAGATTCCGTTC-3’

Mouse Sox4 R 5’-GGTGCCCGACTTCACCTTC-3’

Mouse Sox7 F 5’-ATGCTGGGAAAGTCATGGAAG-3’

Mouse Sox7 R 5’-CGTGTTCTGGTCACGAGAGA-3’

Mouse Sox9 F 5’-AGTACCCGCATCTGCACAAC-3’

Mouse Sox9 R 5’-ACGAAGGGTCTCTTCTCGCT-3’

Mouse Sox17 F 5’-GATGCGGGATACGCCAGTG-3’

Mouse Sox17 R 5’-CCACCTCGCCTTTCACCTTTA-3’

Mouse Sox18 F’ 5’-CCTGTCACCAACGTCTCGC-3’

Mouse Sox18 R 5’- GCAACTCGTCGGCAGTTTG-3’
